# Diet of the Japanese

**Published:** 1882-02

**Authors:** 


### DIET OF THE JAPANESE.

OF thirty-six thousand cows slaughtered in Japan last year, more than one-half were consumed by foreigners on shore or ship. Few natives, except officers in the capital, sailors and soldiers, eat beef. Mutton and pork beyond the treaty ports are hardly yet known. About two hundred varieties of fish are eaten, one-half of the people eating fish every day. The food of the masses is "ninety per cent. vegetable." The list of food-plants in use, with their analysis, not including sea plants, was prepared for a pamphlet by Prof. Edward Kinch, of the Tokio University. A large number of these substances are unknown, or at least unused, in the United States. Of rice, which occupies in its culture one-half of the cultivated land, there are two hundred and fifty varieties of seed in the country. Millet is extensively used, but bread raised from a "sponge" of yeast is hardly yet known in the popular diet, the old Latin-Portuguese word *pan* being, however, in use. The soy-bean, which in chemical composition closely approaches animal fibre, is extensively cultivated. Probably no country excels Japan in the variety of leguminous plants raised for food. Of tubers and roots, the sweet potato is the most popular, though strange to say, as much tabooed by the aristocratic classes as onions are supposed to be among us. Sixteen million bushels of these "Satsuma potatoes" were produced last year, while the "Java" or "Dutch"—our common white potato—is left to foreigners, the native palate not liking it. Lily bulbs—sixteen varieties—serve as food, boiled and served with "drawn butter." The lotus root is eagerly eaten without oblivion of country or decay of patriotism. Poppy seeds powdered as condiment, infusions of salted cherry blossoms for drink, horse chestnuts and acorns are among the articles of diet.

---

"PAT, my boy, we must all of us die once." The sick man turned over in a disgusted frame of mind and replied: "That's just what bothers me. If we could only die half a dozen times I wouldn't worry about this."

A RURAL subscriber wants to know if it makes any difference in the lastingness of fence posts, whether you set them "top end up," the same way the tree grew, or "top end down." In setting a hen, however, there is a vital importance in this distinction, which the careful poulterer will do wisely to observe.—*Hawkeye.*

